# Three-dimensional direct measurement of cardiomyocyte volume, nuclearity, and ploidy in thick histological sections

**DOI:** 10.1038/srep23756

**Published:** 2016-04-06

**Authors:** Jonathan Guy Bensley, Robert De Matteo, Richard Harding, Mary Jane Black

**Affiliations:** 1 Department of Anatomy and Developmental Biology, School of Biomedical Sciences, Monash University, Clayton, Victoria, 3800, Australia

## Abstract

Quantitative assessment of myocardial development and disease requires accurate measurement of cardiomyocyte volume, nuclearity (nuclei per cell), and ploidy (genome copies per cell). Current methods require enzymatically isolating cells, which excludes the use of archived tissue, or serial sectioning. We describe a method of analysis that permits the direct simultaneous measurement of cardiomyocyte volume, nuclearity, and ploidy in thick histological sections. To demonstrate the utility of our technique, heart tissue was obtained from four species (rat, mouse, rabbit, sheep) at up to three life stages: prenatal, weaning and adulthood. Thick (40 μm) paraffin sections were stained with Wheat Germ Agglutinin-Alexa Fluor 488 to visualise cell membranes, and DAPI (4′,6-diamidino-2-phenylindole) to visualise nuclei and measure ploidy. Previous methods have been restricted to thin sections (2–10 μm) and offer an incomplete picture of cardiomyocytes. Using confocal microscopy and three-dimensional image analysis software (Imaris Version 8.2, Bitplane AG, Switzerland), cardiomyocyte volume, nuclearity, and ploidy were measured. This method of staining and analysis of cardiomyocytes enables accurate morphometric measurements in thick histological sections, thus unlocking the potential of archived tissue. Our novel time-efficient method permits the entire cardiomyocyte to be visualised directly in 3D, eliminating the need for precise alignment of serial sections.

Cardiomyocytes are the individual functional units of cardiac muscle, providing the contractile power of the heart. Development of cardiac muscle is a far more dynamic process than previously realised; in humans, the total number of cardiomyocytes triples between birth and 20 years of age^1^. Cardiomyocytes are replaced at a rate of 5% per year at 15 years and 0.5% at 60 years^2^; however, the additional and replacement cardiomyocytes do not have the capacity to repair the heart after injury^3^. Given that abnormal growth of the myocardium, particularly ventricular hypertrophy, is strongly linked to adverse cardiovascular events in later life^4^, it is important to understand how cardiac muscle grows in early life and how it responds to cardiovascular disease and injury.

In order to characterise the growth and properties of individual cardiomyocytes within the myocardium there are currently two methodological procedures that can be employed; one is to enzymatically isolate cardiomyocytes from fresh unfixed heart tissue using a collagenase enzyme^5^ and the other is to fix and embed tissue samples in paraffin, OCT (for frozen sectioning), or resin.

Enzymatically isolated cardiomyocytes can be used for the measurement of cell volume, nuclearity (number of nuclei per cell), and ploidy (number of genome copies per nucleus). Enzymatic isolation is essential for studies employing cell culture, flow cytometry, or *ex-vivo* analysis of calcium signalling by confocal microscopy. Obtaining enzymatically isolated cells usually requires fresh cardiac tissue. Not only does this approach prevent the examination of the cardiomyocytes *in situ*, but there is also the possibility that the isolation process alters cell dimensions and volume. Cellular isolation from fixed, archival tissue is difficult. It is possible to isolate cells from fixed cardiac tissue, using 12.5 mol/L aqueous potassium hydroxide^6^; however, the yield is low and the data generated does not match that obtained from enzymatically isolated cardiomyocytes from unfixed fresh tissue^7^. Furthermore, this method can lead to the destruction of smaller mononucleated cardiomyocytes^7^.

Cardiac tissue is usually archived as tissue samples embedded in paraffin wax. Typically, the analysis of heart tissue from paraffin blocks has been restricted to thin sections (4–7 μm), which provides only a restricted view of cardiomyocytes. As cardiomyocytes are differentially oriented, depending on where they are located in the heart, analysis of thin sections can result in inaccurate morphometric measurements. To date, the most common approach to assessing cardiomyocyte dimensions or volume in paraffin sections has been to measure the cross–sectional area of the cardiomyocyte; however, there are limitations with the use of cross-sectional area as a surrogate measure of cardiomyocyte volume. Cross sectional area around the short axis of the cardiomyocyte presents only a 2-dimensional picture of a 3 dimensional cell; importantly it ignores the length of the cardiomyocyte, which can vary considerably within the same heart, and is unable to account for cardiomyocytes that branch (a common characteristic of differentiated cardiomyocytes)^8^,^9^,^10^. Other researchers have used thin serial sections (4–7 μm), followed by immunohistochemistry to identify cell boundaries, and then re-constructed the images to measure cell volume^11^. This method is also predicated on the serial sections consistently being of identical thickness, which is technically challenging.

Measuring cardiomyocyte nuclearity is also important in studies of myocardial development and disease. In many species, binuclearity is a mark of cardiomyocyte maturity^12^ (but not in humans or other primates^13^). Another important measure is ploidy, especially in humans^2^ and other primates in which the majority of cardiomyocytes remain mononucleated throughout life, with these mononucleated cardiomyocytes usually becoming tetraploid (4n) during adolescence^1^. Cardiomyocyte polyploidy is often an indicator of stress or abnormal cardiomyocyte development and maturation^12^.

Recent studies suggest that abnormalities in cardiac growth may have their origins in early life. Much of our knowledge of myocardial development has been derived from animal studies^10^,^14^,^15^,^16^,^17^,^18^. The applicability to humans of findings from animal models is dependent on how closely the growth of cardiomyocytes in the animal model resembles that in the human heart. In order to determine how prenatal, perinatal, and postnatal growth of cardiomyocytes in a given species resembles that in the human, it is imperative to fully characterise the normal growth of cardiomyocytes in that species.

Owing to shortcomings with existing morphometric methods for analysing cardiac muscle, our aim was to develop an accurate, time-efficient, and cost-efficient method for analysing cardiomyocyte growth (size, nuclearity, and ploidy) in paraffin-embedded samples from different species at different life stages. This technique employs a lectin, wheat germ agglutinin, and extends the use of this lectin to whole thick sections. Importantly, this technique permits direct measurements to be made without reconstructions. The quantitative approach we describe is novel although it draws on established methods; our new approach is robust and can be readily used by researchers to advance our understanding of cardiomyocyte growth and disease processes.

## Results

Utilising established techniques (described in the Methods section) we have developed a novel approach to the concomitant measurement of cardiomyocyte volume, ploidy, and nuclearity within fixed heart samples from different species (mice, rabbits, rats, and sheep). The approach that we have developed and the subsequent data generated are detailed in this section. Using this approach we have been able to simultaneously measure cardiomyocyte volume, ploidy, and nuclearity in up to 400 cells per day.

### Orientation of cardiomyocytes in the longitudinal axis

**Figure 1:**
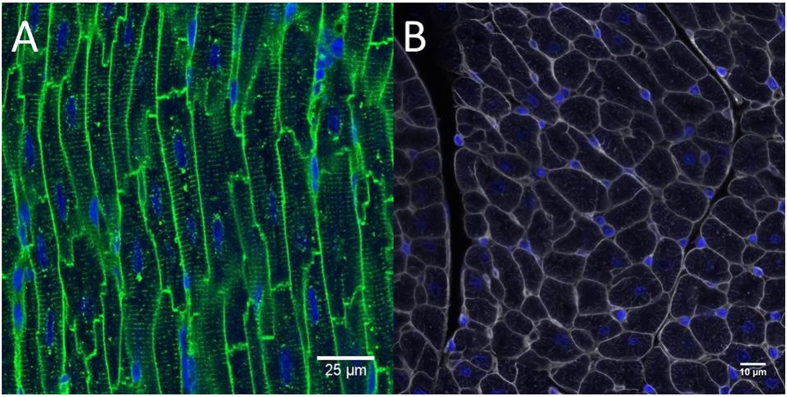
(**A**) Cardiomyocytes in long section, DAPI appears in blue, WGA-AF488 appears in green. (**B**) Cardiomyocytes in cross-section, DAPI appears in blue, WGA-AF488 appears in grey.

Prior to cutting thick (40 μm) sections, a 4–5 μm paraffin section was cut and stained with haematoxylin and eosin, in order to determine the orientation of cardiomyocytes within the tissue. The desired orientation is with the long axis of the cardiomyocytes in the cutting plane so that all nuclei within each cardiomyocyte could be visualised. If only the short axis was present, the specimen was re-embedded at 90 degrees to the original cutting plane to align the cardiomyocytes in the longitudinal plane (Fig. 1A).

### Image acquisition

Image acquisition was performed using either a Leica SP5 confocal microscope (Leica Microsystems, Germany) or a Nikon C1 confocal microscope (Nikon, Japan).

When using a Leica confocal microscope, which was equipped with LAS AF software (Leica Application Software–Advanced Fluorescence), we performed compensation on a z-stack acquisition using the Photomultiplier Tube (PMT) method and/or the Acousto-Optical Tuneable Filter (AOTF) method. The PMT method regulates the voltage within the PMT; this voltage is the acceleration energy (the gain) applied to the electrons within the PMT. In the AOTF method, a filter is used to regulate the amount of laser light delivered to the specimen, without changing the power of the laser itself (which is usually constant). In setting the AOTF method, the amount of laser light is set in a range from 0 to 100% (100% is the maximum rated power of the laser that is allowed to pass through the filter).

When using a Nikon confocal microscope, we used the Z intensity correction feature to perform the acquisition in NIS-Elements (Nikon, Japan). The optical design and software in other confocal microscopes (e.g. Bio-Rad, Zeiss) are different from those in Leica and Nikon microscopes; if using these other microscopes it would be necessary to obtain instructions from the microscope’s manufacturer on how to perform an intensity correction.

**Figure 2:**
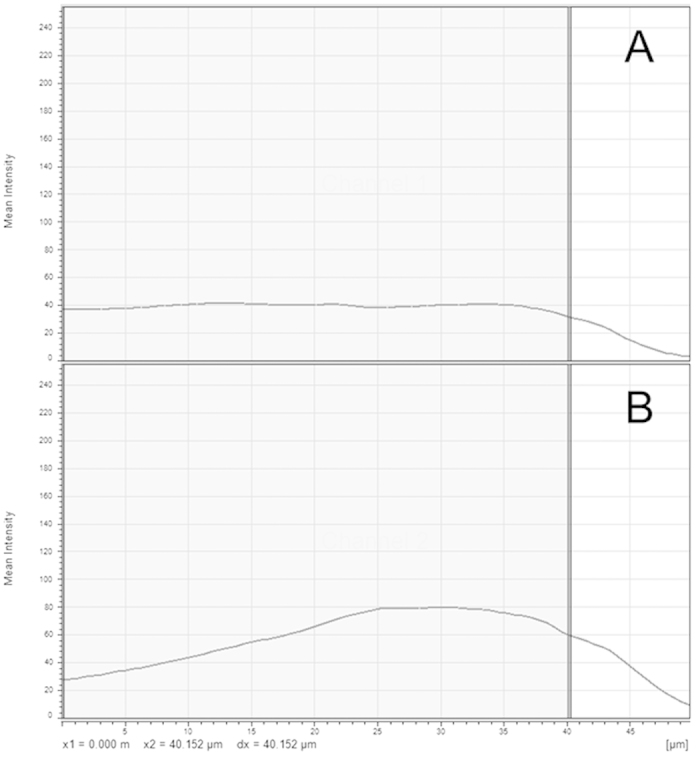
(**A,B**) Representative mean intensity chart. (**A**) represents the mean intensity through the z-stack for DAPI. (**B**) represents the mean intensity through the z-stack for WGA-AF488. The X axis represents the depth through the section (z-stack), the Y axis is the mean intensity per channel at each depth (frame of the z-stack).

When performing ploidy analysis, we found it essential that the brightest pixels in the acquisition were not close to the saturation limit of the PMT and were above the noise floor. Signal intensity is crucial to measuring ploidy accurately: a signal that is too dim will be indistinguishable from background noise, whereas a signal at the limit of detection eliminates dynamic range; for example a signal 4 times the detector upper limit will appear the same as a signal 20 times the detector upper limit. The appearance of cells with the desired DAPI intensity is shown in Fig. 1A,B. The mean intensity through the z-stack is flat, with a ‘drop-off’ noted as the section ends (on the right-hand side of the chart). When this “drop-off” was not achieved, nuclei deeper in the tissue appeared dimmer than in reality and nuclei closer to the objective lens appeared brighter than they actually were. For the image shown in Fig. 2A, the signal intensity at no point reaches zero and is not near the intensity limit. Ploidy controls were acquired using identical settings to cardiomyocyte acquisitions and were included in the analysis with the nuclei channel (DAPI) only. The intensity profile for WGA-AF488 is shown in Fig. 2B; intensity linearity was not required as this channel was not quantified and was optimised for image analysis performance. The image acquisition goal for the WGA-AF488 channel is to provide thin and well separated cardiomyocyte membranes.

### Cardiomyocyte analysis

**Figure 3:**
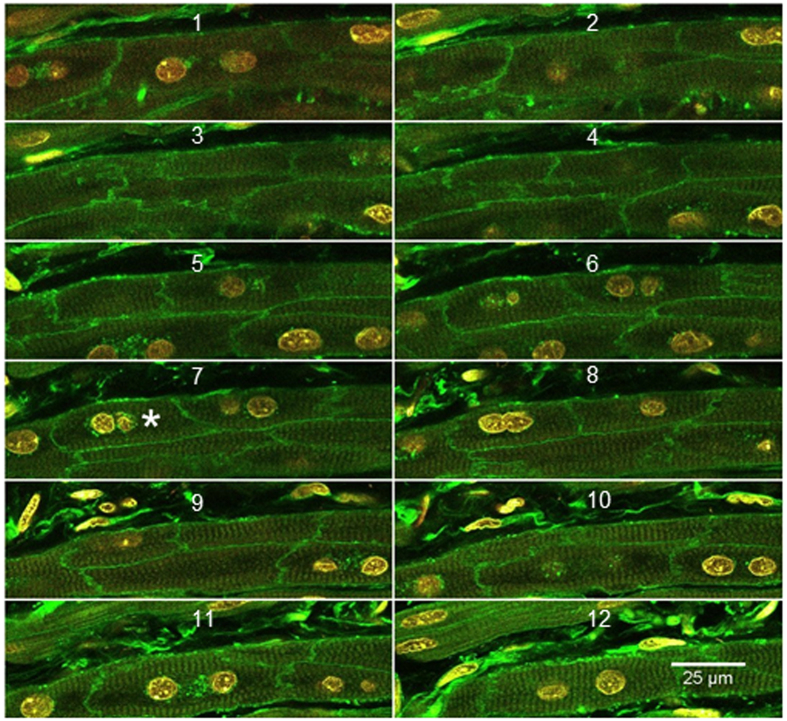
Cardiomyocyte example. 3.5 μm pitch across 12 sections (42 μm coverage). DAPI appears in yellow/red, WGA-AF488 appears in green. *denotes the example cell analysed in Fig. 4. Scale bar represents 25 μm.

Cardiomyocytes within the sections were easily recognisable by their cytoplasmic striations (Fig. 3). We used the Imaris Version 8.2 module, called ImarisCell, for our analysis. We provide our method here, but this technique can also be conducted using the user’s manual for Imaris. In ImarisCell, the membrane staining option was chosen and the nuclear channel (DAPI) was used as the seed for growth to the limits of the membrane staining. The ImarisCell single nucleus per cell option was not used; however, nuclei not surrounded by cytoplasm were excluded from the analysis. Each measurement of cell volume was checked manually to ensure the cell was delineated normally, with complete or almost complete membrane staining. If necessary, cardiomyocytes were split manually to correct for abnormal seed growth, incomplete membrane staining, or abnormal membrane staining. Partial or incomplete cells were manually removed from the analysis. Cell volume, nuclear volume, nuclearity (number of nuclei per cell) and integrated intensity of each nucleus (ploidy) were calculated by ImarisCell. The most important aspect of the analysis process is consistency during image acquisition and analysis, and awareness of several factors related to the tissue. If the PMT voltage or laser power is too high, this will make the membranes appear thicker than they actually are, thereby making the cells appear smaller than they actually are. When there is fibrosis or other pathology, excess collagen or laminin around the cardiomyocyte may make the membrane appear thicker than in reality. In embryos and young animals/humans, there is minimal collagen/laminin around each cardiomyocyte; this requires careful attention to balance the desire to acquire complete and thick cell membranes to facilitate easier image analysis, versus the more challenging task of reliably analysing very thin membranes with little collagen/laminin, with possible incomplete staining. Unfortunately, currently available super-resolution microscopes lack the ability to penetrate deep within tissue sections and resolve such fine details.

### Measurement repeatability and time

8,900 randomly selected cardiomyocytes were analysed more than once to determine the repeatability of the cardiomyocyte volume measurements. The difference in cardiomyocyte volume between the first and second measurements of each individual cardiomyocyte was 6.56 ± 2.10% (mean ± SD). We estimate that 300–400 cardiomyocytes can be analysed per day, even by inexperienced researchers. However, this will depend on tissue quality, staining performance, and image acquisition factors.

### Analysis alternatives

**Figure 4:**
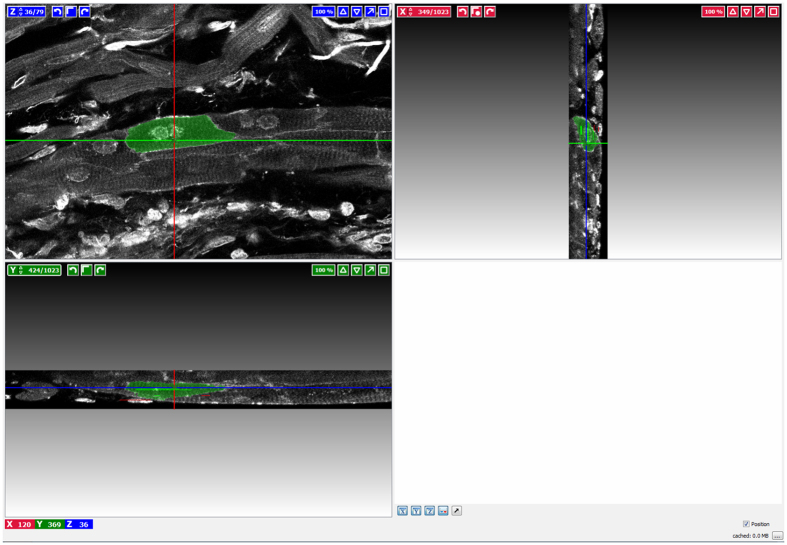
Cardiomyocyte delineation example. The selected cardiomyocyte is in green. In the top left hand corner is the Z-stack position, in the top right hand corner is the X axis projection. In the bottom left hand corner is the Y axis projection. Seed was laid down at the cross-hair point.

**Figure 5:**
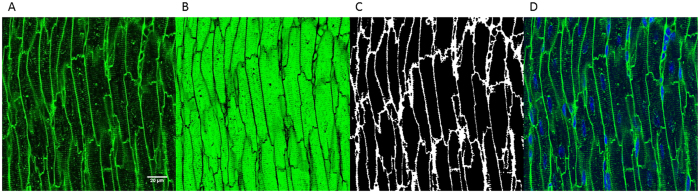
Alternative method for cardiomyocyte volume measurement. In (panel **A**) WGA-AF488 staining appears in green. (Panel **B**) shows a brightness inversion of the left panel. In (panel **C**) the image was binarised and manual clean-up performed to produce dark cytoplasm and bright membranes. In (panel **D**) the composite image including both the WGA-AF488 (again in green) and DAPI is shown (in blue).

Imaris is an expensive software application and may not be affordable by many laboratories. As an alternative to analysis with Imaris, other 3-dimensional software (e.g. Amira, Volocity, FIJI, or Huygens) can be used but this requires some modifications in the analysis procedures. When analysing z-stacks using Volocity, Amira, FIJI, or Huygens, we found it preferable to perform a brightness inversion on the WGA-AF488 channel to produce a bright cytoplasm with dark membranes (or dark cytoplasm and bright membranes) (Fig. 4). The brightness inversion required some manual ‘clean-up’ to produce clear membranes which facilitated the analysis of cardiomyocyte volume. It was necessary that the ‘fill holes’ option was enabled when estimating the final cardiomyocyte volumes, to create a single object without internal holes. See Video 1 (supplementary data) for a 3-dimensional representation of the usual appearance of the left ventricle. It is also possible to use cardiomyocyte objects created in ilastik via FIJI (a distribution of ImageJ), as show in green in Fig. 5. The production of these objects is essentially in the form of a z-stack of images with either cell (positive) or background (negative) present. This z-stack of images then replaces the WGA channel acquired on the confocal microscope.

### Cardiomyocyte volume, nuclearity, and ploidy

To demonstrate the utility of our novel approach, ventricular cardiomyocytes were analysed from the hearts of four species at different life stages; a total of 34 animals was used. Table 1 describes the data obtained from each species and age, stratified by ventricle; (left ventricle with septum (LV + S), and right ventricle (RV)), and nuclearity (mononucleated, binucleated).

### Other cellular events

**Figure 6:**
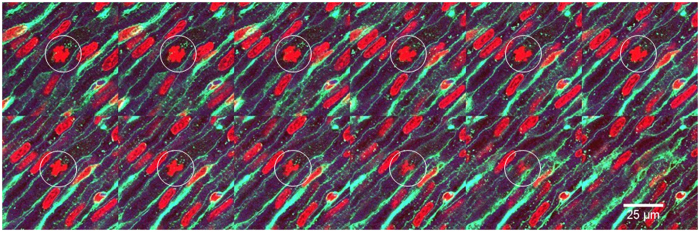
Z-stack through the left ventricle of a fetal sheep. 333 nm pitch across 4 μm of total coverage. DAPI appears in red, WGA-AF488 appears in green. Mitotic nucleus appears in the white circles.

The technique we describe enables the identification of cellular events in individual cells such as mitosis, apoptosis, and sarcomere disorganisation. For example, a mitotic cardiomyocyte nucleus from the left ventricle of a fetal sheep is shown in Fig. 6, with the mitotic nucleus circled in white.

### Biological Results

#### Mouse

The majority of cardiomyocytes in weanling mice were binucleated (LV + S: 80 ± 1.4%, RV: 85.1 ± 1.4%). Binucleated cardiomyocytes had larger average volumes than mononucleated cardiomyocytes. In the LV + S, binucleated cardiomyocytes were 42.1% larger in volume than mononucleated cardiomyocytes, whereas in the RV the binucleated cardiomyocytes were 122% larger in volume than mononucleated cardiomyocytes. Right ventricular cardiomyocytes were substantially larger in volume than those from the LV + S (both mononucleated and binucleated), which may reflect the right ventricular dominance before birth^19^. No tetraploid (4n) cardiomyocyte nuclei were observed.

Cardiomyocytes from adult mice were predominantly binucleated in both ventricles (LV + S 95.6 ± 0.4%, RV: 96.2 ± 0.9%). In the LV + S, the binucleated cardiomyocytes were 41.5% larger in volume than mononucleated cardiomyocytes, whereas in the RV the binucleated cardiomyocytes were 15.5% larger in volume than mononucleated cardiomyocytes. Tetraploid cardiomyocyte nuclei were present in both ventricles, and in both mononucleated (1 nucleus of 4n) and binucleated (2 nuclei of 4n each) cardiomyocytes, albeit at relatively low rates and with high intra-mouse variability.

#### Rabbit

In weanling rabbits, the majority of cardiomyocytes in both ventricles were binucleated (LV + S: 86.4 ± 1.1%, RV: 86 ± 3.7%), with no evidence of tetraploidy. In the LV + S, binucleated cardiomyocytes were 48.6% larger in volume than mononucleated cells, whereas in the RV, binucleated cardiomyocytes were 69.4% larger in volume than mononucleated cardiomyocytes. Overall, right ventricular cardiomyocytes (mononucleated and binucleated) were significantly larger in volume than those from the LV + S.

#### Rat

The majority of cardiomyocytes in the adult rat heart were binucleated (LV + S: 96.5 ± 0.3%, RV: 96.8 ± 1.1%). However, 7–10% of cardiomyocytes from both ventricles had tetraploid nuclei; mononucleated (1 nucleus of 4n), and binucleated (2 nuclei of 4n each) cardiomyocytes, with low variability between animals in the numbers of 4n nuclei found. In the LV + S, binucleated cardiomyocytes were 41.4% larger in volume than mononucleated cells, whereas the RV binucleated cardiomyocytes were 34.5% larger in volume than mononucleated cells.

#### Sheep

The fetal sheep hearts (at approximately 0.9 of term) contained mostly binucleated cardiomyocytes (LV + S: 87.1 ± 1.6%, RV: 91.1 ± 1.2%). RV binucleated cardiomyocytes were 92.3% larger in volume than mononucleated cells and LV + S binucleated cardiomyocytes were 61.6% larger in volume than mononucleated cells. All fetal cardiomyocytenuclei that were analysed were diploid.

In 9 week old lambs, binucleated cardiomyocytes predominated (LV + S: 97.6 ± 0.9%, RV: 98.4 ± 0.7%), and the proportion of these had not changed in adulthood (LV + S: 98.5 ± 1.5%, RV: 98.5 ± 0.3%). At 9 weeks a small proportion of RV cardiomyocytenuclei were tetraploid (0.1 ± 0.1%). In adult sheep, tetraploidy was variably present in mononucleated cells (one of the two adult sheep had no tetraploidy), but was present in 2–10% of binucleated cardiomyocytes (2 nuclei of 4n each) in both adult sheep.

### Assessment of myocardial capillarisation

**Figure 7:**
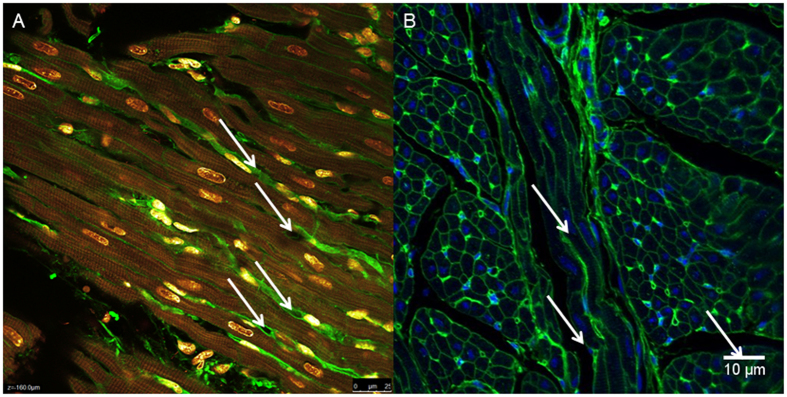
(**A,B**) Identification of capillaries by WGA-AF488 enhancing staining. On the left (Panel **A**) DAPI appears in yellow/red, WGA-AF488 appears in green. On the right (Panel **B**) DAPI appears in blue, WGA-AF488 appears in green. Arrows identify capillaries.

**Figure 8:**
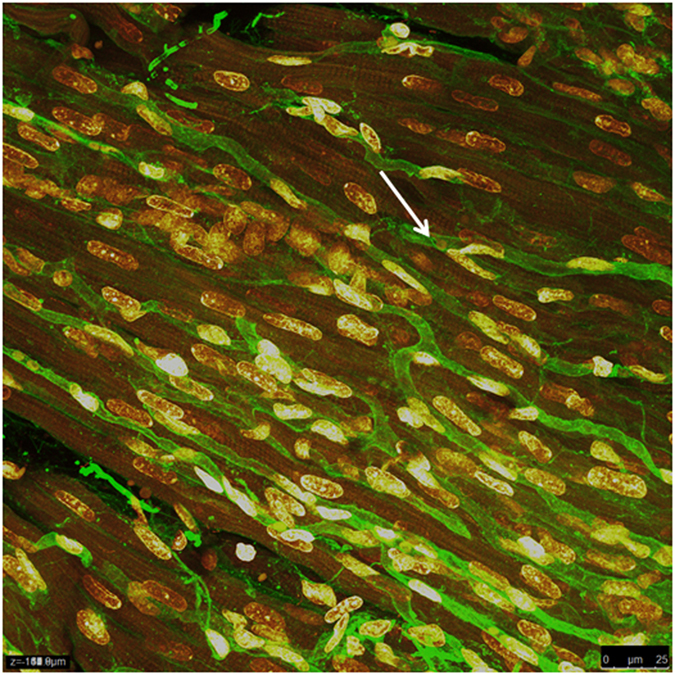
Brightest point projection method to demonstrate the 3-dimensional appearance of capillaries in the heart, from the same z-stack as Fig. 7A.

In addition to staining cellular membranes, the fluorescently labelled WGA-AF488 also clearly binds to blood vessels because endothelial cell membranes have a high content of sialic acid, particularly on the apical surface (Fig. 7A,B). Hence, using the described staining approach, it is possible to accurately identify blood capillaries and would therefore be possible to make measurements such as capillary density, capillary length and surface area, and diffusion radius^20^,^21^. The relationship between cardiomyocytes and capillaries can be clearly observed in 3-D space (Fig. 8). We have recently reported the use of wheat germ agglutinin in the quantitative assessment of capillarisation by measuring the number of capillary profiles relative to the number of cardiomyocyte profiles in the fetal sheep heart^22^.

## Discussion

In this study, we have developed and optimised a technique permitting the direct and simultaneous analysis of cardiomyocyte volume, nuclearity, and ploidy in 3-dimensional z-stack images using thick histological sections. When first using our approach, the degree of repeatability can be assessed by staining one section of heart muscle, completing the analysis and repeating the analysis again; a variation of <5–10% is achievable and acceptable.

Although we have included the results of our analysis solely as a demonstration of the utility of our technique, we found that the sizes of cardiomyocytes from the four species analysed are very similar, thereby confirming previous findings^23^.

In a previous publication in which we reported the effects of preterm birth on the development and maturation of cardiomyocytes in sheep, we used an earlier version of the method described here to measure cardiomyocyte volume, nuclearity, and ploidy^12^. To our knowledge, this was the first published method to measure cardiomyocyte volume, nuclearity, and ploidy in 3 dimensions directly from thick paraffin sections, without serial sections or reconstructions. This earlier version utilised carboxymethylcellulose in the staining solution to inhibit over-staining, ≥100 μm paraffin sections to capture more cells in one section, and a multi-photon laser to obtain sufficient light penetration into the tissue^12^. Cutting paraffin sections at ≥100 μm is challenging and obtaining a good section is very difficult. The method we describe here eliminates these restrictions and enables this method to be performed in a cost and time efficient manner; it has the added advantage that it does not require a multi-photon laser.

## Conclusion

Our method allows for the direct simultaneous quantitative analysis of cardiomyocyte volume, nuclearity, and ploidy in thick sections, eliminating the need for serial sectioning and image reconstruction. This technique can be readily used to comprehensively analyse the properties of individual cardiomyocytes, and is particularly useful when analysing archived samples of heart tissue.

## Methods

Here we briefly describe the basic techniques used to measure the volume, nuclearity, and ploidy of cardiomyocytes in thick paraffin sections. The Results section provides a detailed explanation of the analysis undertaken.

### Ethical approval

We used archived fixed heart tissue from previous experiments, all of which were approved by the Monash University Animal Ethics Committee (Monash University, Victoria, Australia). These previous experiments were conducted in accordance with the National Health and Medical Research Council (Australia) guidelines on the care and use of animals for scientific purposes (National Health and Medical Research Council, Canberra, Australia).

### Cardiac muscle samples

Heart muscle from the RV, and the LV + S were analysed separately. Samples used for analysis were taken from formalin-fixed paraffin embedded hearts from: 10 weanling and 3 adult mice, 4 weanling rabbits, 3 adult rats, 7 fetal sheep (at ~131 days gestation, with ~147 days as term), 5 nine-week-old lambs, and 2 adult sheep. Weanling mouse and rabbit hearts had been immersion fixed in formaldehyde solution. All sheep hearts, adult mouse, and adult rat hearts had been perfusion fixed via the aorta with formaldehyde solution. Tissue was processed into paraffin using a Leica Peloris II tissue processor (Melbourne, Australia).

### Tissue sectioning and staining

40 μm paraffin sections were cut and placed onto SuperFrost Plus slides (Menzel, Germany). Using Wheat Germ Agglutinin–Alexa Fluor 488 (Invitrogen, USA) and DAPI (4,6-diamidino-2-phenylindole hydrochloride, Invitrogen, USA), these sections, along with the ploidy controls on the same slide, were stained in a Coplin jar utilising the protocol described in Table 2. The ploidy control was a semen spot (from the same species as the heart tissue) placed on the opposite end of the slide, or where semen was not available, a paraffin section of testis was used. Sections were mounted using ProLong Gold (Invitrogen, USA). Sections were protected from light during staining and were stored in the dark at room temperature after staining.

### Confocal microscopy

Confocal z-stacks were captured using either a Leica SP5 confocal microscope (Leica Microsystems, Germany) or a Nikon C1 confocal microscope (Nikon, Japan). 100x and 63x oil immersion objectives were utilised for all acquisitions. Deconvolution was not performed on any images.

### Image Analysis measurements of cardiomyocyte volume, ploidy, and nuclearity

Cardiomyocyte volume and ploidy were measured in z-stacks using Imaris Version 8.2 software (Bitplane AG, Switzerland); the number of nuclei within each cell was also recorded.

## Additional Information

**How to cite this article**: Bensley, J. G. *et al.* Three-dimensional direct measurement of cardiomyocyte volume, nuclearity, and ploidy in thick histological sections. *Sci. Rep.* **6**, 23756; doi: 10.1038/srep23756 (2016).

## Supplementary Material

Supplementary Information

Supplementary Video 1

## Figures and Tables

**Table 1:** Summary of cardiomyocyte data from each species and life stage.

Species	Left Ventricle with Septum						Right Ventricle					
	MononucleatedPercentage	MononucleatedVolume(µm^3^)	MononucleatedPloidy (n)	BinucleatedPercentage	BinucleatedVolume (µm^3^)	BinucleatedPloidy (n)	MonucleatedPercentage	MononucleatedVolume(µm^3^)	MononucleatedPloidy (n)	BinucleatedPercentage	BinucleatedVolume(µm^3^)	BinucleatedPloidy (n)
Weanling Mouse(n = 10) 8,923 cells	20 ± 1.4%	5,634 ± 1,035	2n:100 ± 0%	80 ± 1.4%	8,007 ± 1404	2*2n:100 ± 0%	14.9 ± 1.4%	6,651 ± 1,666	2n:100 ± 0%	85.1 ± 1.4%	14,763 ± 3,405	2*2n:100 ± 0%
Adult Mouse(n = 3) 2,007 cells	4.4 ± 0.4%	16,851 ± 403	2n:97.7 ± 1.5%4n:2.3 ± 1.5%	95.6 ± 0.4%	23,845 ± 186	2*2n:91.3 ± 5.9%2*4n:8.7 ± 5.9%	3.8 ± 0.9%	13,116 ± 811	2n:98 ± 2%4n:2 ± 2%	96.2 ± 0.9%	15,154 ± 1,045	2*2n:95.3 ± 2.4%2*4n:4.7 ± 2.4%
Weanling Rabbit(n = 4) 3,200 cells	13.6 ± 1.1%	6,063 ± 529.5	2n:100 ± 0%	86.4 ± 1.1%	9,015 ± 553	2*2n:100 ± 0%	14 ± 3.7%	6,699 ± 611	2n:100 ± 0%	86 ± 3.7%	11,347 ± 758	2*2n:100 ± 0%
Adult Rat(n = 3) 5,211 cells	3.5 ± 0.3%	18,006 ± 541	2n:93 ± 0.6%4n:7 ± 0.6%	96.5 ± 0.3%	25,453 ± 117	2*2n:90 ± 1.5%2*4n:10 ± 1.5%	3.2 ± 1.1%	16,100 ± 952	2n:92.3 ± 1.5%4n:7.7 ± 1.5%	96.8 ± 1.1%	21,662 ± 852	2*2n:90.7 ± 0.7%2*4n:9.3 ± 0.7%
Fetal Sheep(n = 7) 8,000 cells	12.9 ± 1.6%	3,417 ± 1,169	2n:100 ± 0%	87.1 ± 1.6%	5,522 ± 1,221	2*2n:100 ± 0%	8.9 ± 1.2%	5,278 ± 909	2n:100 ± 0%	91.1 ± 1.2%	10,152 ± 687	2*2n:100 ± 0%
9 week old Lamb(n = 5) 28,103 cells	2.4 ± 0.9%	9,998 ± 648	2n:100 ± 0%	97.6 ± 0.9%	18,344 ± 685	2*2n:100 ± 0%	1.6 ± 0.7%	10,162 ± 462	2n:99.9 ± 0.1%4n:0.1 ± 0.1%	98.4 ± 0.7%	13,164 ± 246	2*2n:100 ± 0%
Adult Sheep(n = 2) 2,938 cells	1.5 ± 1.5%	19,942 ± 2,732	2n:97.4 ± 2.6%4n:2.6 ± 2.6%	98.5 ± 1.5%	27,111 ± 4,923	2*2n:92 ± 2%2*4n:8 ± 2%	1.5 ± 0.3%	14,549 ± 904	2n: 99.0 ± 1%4n:1 ± 1%	98.5 ± 0.3%	18,051 ± 401	2*2n:95 ± 32*4n:5 ± 3

Data are segregated as Left Ventricle with Septum and Right Ventricle, and are further divided into mononucleated and binucleated cardiomyocytes. n = refers to the number of animals; number of cells is the total number of cells analysed.

**Table 2:** Recommended staining protocols for heart issue on glass slides (4 to 40 μm sections).

	4–8 μm Paraffin Sections	40 μm Paraffin Sections
Dewax and rehydration	3 changes of 2 minutes each–Xylene3 changes of 2 minutes each-Ethanol	3 changes of 10 minutes each–Xylene 3 changes of 10 minutes each-Ethanol
Wash	2 minutes in running water*	10 minutes in running water*
Stain	10 μg/mL WGA-AF488 and1 μg/mL DAPI in PBS or HBSS 15 minutes	5 μg/mL WGA-AF488 and 1 μg/mL DAPI in PBS or HBSS 4 hours to overnight
Wash	2 changes of 2 minutes each in PBS or HBSS	3 changes of 5 minutes each in PBS or HBSS
Mount	ProLong Gold or similar	ProLong Gold or similar

WGA-AF488 = Wheat Germ Agglutinin-Alexa Fluor 488 (Invitrogen, USA). *It is acceptable to wash for longer without adverse effects.
