# Supplementary material for: Three-dimensional direct measurement of cardiomyocyte volume, nuclearity, and ploidy in thick histological sections
Source: Sci Rep. 2016 Apr 6;6:23756. doi: 10.1038/srep23756 (PMC4822151; doi:10.1038/srep23756)
Supplement: Supplementary Information [file srep23756-s1.doc]

Three-dimensional direct measurement of cardiomyocyte volume, nuclearity, and ploidy in thick histological sections

Jonathan Guy Bensley, Robert De Matteo, Richard Harding, Mary Jane Black*

Supplementary Information

Video 1: Confocal z-stack of cardiomyocytes. Wheat Germ Agglutinin-Alexa Fluor 488 appears in green. DAPI (nuclei) appear in yellow/red. Total z coverage is 46µm, with 666nm pitch. This video is from the same z-stack series that appears in Figure 3.
